# Influence of metabolic stress and metformin on synaptic protein profile in SH‐SY5Y‐derived neurons

**DOI:** 10.14814/phy2.15852

**Published:** 2023-11-27

**Authors:** Alex J. T. Yang, Ahmad Mohammad, Michael S. Finch, Evangelia Tsiani, Gaynor Spencer, Aleksandar Necakov, Rebecca E. K. MacPherson

**Affiliations:** ^1^ Department of Health Sciences, Faculty of Applied Health Sciences Brock University St Catharines Ontario Canada; ^2^ Department of Biological Sciences Brock University St Catharines Ontario Canada; ^3^ Centre for Neuroscience Brock University St. Catharines Ontario Canada

**Keywords:** Alzheimer's disease, AMPK, insulin‐resistance, metformin, neuronal health

## Abstract

Insulin resistance (IR) is associated with reductions in neuronal proteins often observed with Alzheimer's disease (AD), however, the mechanisms through which IR promotes neurodegeneration/AD pathogenesis are poorly understood. Metformin (MET), a potent activator of the metabolic regulator AMPK is used to treat IR but its effectiveness for AD is unclear. We have previously shown that chronic AMPK activation impairs neurite growth and protein synthesis in SH‐SY5Y neurons, however, AMPK activation in IR was not explored. Therefore, we examined the effects of MET‐driven AMPK activation with and without IR. Retinoic acid‐differentiated SH‐SY5Y neurons were treated with: (1) Ctl: 24 h vehicle followed by 24 h Vehicle; (2) HI: 100 nM insulin (24 h HI followed by 24 h HI); or (3) MET: 24 h vehicle followed by 24 h 2 mM metformin; (4) HI/MET: 24 h 100 nM insulin followed by 24 h 100 nM INS+2 mM MET. INS and INS/MET groups saw impairments in markers of insulin signaling (Akt S473, mTOR S2448, p70s6k T389, and IRS‐1S636) demonstrating IR was not recovered with MET treatment. All treatment groups showed reductions in neuronal markers (post‐synaptic marker *HOMER1* mRNA content and synapse marker synaptophysin protein content). INS and MET treatments showed a reduction in the content of the mature neuronal marker NeuN that was prevented by INS/MET. Similarly, increases in cell size/area, neurite length/area observed with INS and MET, were prevented with INS/MET. These findings indicate that IR and MET impair neuronal markers through distinct pathways and suggest that MET is ineffective in treating IR‐driven impairments in neurons.

## Graphical abstract

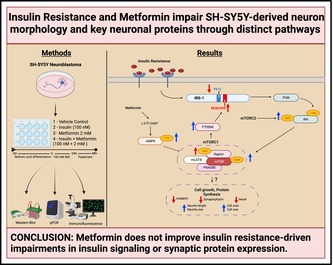

## 1 INTRODUCTION

Alzheimer's disease (AD) is a neurodegenerative dementia that severely impairs cognitive function. AD pathology is traditionally characterized by increased amyloid‐*β* (A*β*) plaque deposits and hyper‐phosphorylated Tau Tangle (Tau) development that are associated with worsening cognitive function (Morris et al., 2014; Zhang et al., 2011). A*β* and Tau have long been the primary focus of AD therapies and treatments, given their robust association with the disease. In the past decade, however, the impact of non‐traditional factors on AD pathology has been described (Mattson & Arumugam, 2018; Morris et al., 2014; Norton et al., 2014; Talbot et al., 2012). Specifically, the role of brain insulin resistance (IR) has emerged as an important contributing factor in the development of AD. As in the periphery, IR in the brain involves the reduced ability of insulin to stimulate its downstream signaling cascade (Folch et al., 2019; Talbot, 2014). Dysregulation of energy homeostasis commonly observed with insulin resistance (IR) and Type‐2 Diabetes Mellitus (T2D) has now been connected to the pathology of AD through molecular signaling and metabolic dysregulation (Chatterjee & Mudher, 2018; Cunnane et al., 2011; Folch et al., 2019; Ho et al., 2004; Mattson & Arumugam, 2018; Willette et al., 2015). Impairments in brain insulin signaling have also been correlated with cognitive decline (Talbot et al., 2012; Walker & Harrison, 2015). Further, brain IR and T2D are marked by synaptic dysfunction and loss (DeKosky & Scheff, 1990; Folch et al., 2019; Spinelli et al., 2019; Talbot et al., 2012; Terry et al., 1991). This synaptic loss is often described as being due to impaired insulin signaling (Folch et al., 2019), such as the hyperphosphorylation of insulin‐receptor substrate (IRS1) at inhibitory serine residues (Chatterjee & Mudher, 2018; Copps & White, 2012; Zhang et al., 2008, 2013). However, it is unclear whether, or how, IR‐driven impairments impact synaptic integrity and function and whether these synaptic impairments are significant in contributing toward the cognitive impairments observed with AD.

Physiological insulin signaling involves the binding of insulin to the insulin receptor, initiating the phosphorylation IRS‐1, leading to recruitment and activation of PI3K, Akt, and finally downstream targets such as the major marker of cell growth and proliferation, the mechanistic target of rapamycin 1 (mTORC1) (Sędzikowska & Szablewski, 2021). Both Akt and mTORC1 are important junctions in the insulin signaling cascade, whereby activation of mTORC1 by Akt in response to insulin drives cell growth and proliferation. Peripherally, reductions in insulin receptor content, hyper‐phosphorylation of inhibitory IRS‐1 serine sites, blunted Akt phosphorylation, and overactive mTORC1 are common impairments observed with T2D and IR (Merz & Thurmond, 2020; Vlavcheski et al., 2020). While these insulin‐driven signaling cascades are present in the brain's response to insulin, the impact of brain IR impairments in these signaling cascades is not well known. Early reports examining post‐mortem AD brains, describe impaired insulin binding (Rivera et al., 2005), reduced insulin receptor content (Steen et al., 2005), and increased inhibitory phosphorylation of various serine sites of IRS‐1 (Moloney et al., 2010). These impairments in insulin signaling have been correlated with worsening episodic and working memory and global cognition scores as AD develops (Talbot et al., 2012) and brain IR has been suggested to be an early process in AD progression (Velazquez et al., 2017). Similarly, hyperactivation of mTORC1 signaling as a result of aberrant Akt signaling seems to occur early in AD development (Tramutola et al., 2015), leading to greater IRS‐1 serine residue phosphorylation (Copps & White, 2012), further impairing insulin signaling (Zhang et al., 2008). IR has been shown to increase A*β*‐plaque development (Franklin et al., 2019; Ho et al., 2004) a hallmark of AD as well as reduce synaptic spine density & synapse formation (Lee et al., 2011). In relation to IR and synaptic markers, IR results in reductions in key synaptic subunits of AMPA and NMDA receptors, impaired synaptic transmission, and worsening spatial memory (Grillo et al., 2015). These results may provide an explanation for the declining cognitive function observed with brain IR development (Talbot et al., 2012). While the impact of peripheral metabolic factors on brain metabolism & cognitive function remains to be explored, these findings have prompted researchers to question whether brain IR and AD can be improved with existing treatments used for peripheral disorders, such as T2D.

Metformin (MET) is the most prescribed T2D drug and effectively treats peripheral metabolic dysregulation (Rena et al., 2017). The proposed pathway by which MET improves metabolic function is by lowering hepatic glucose production (Rena et al., 2017; Wang et al., 2019), improving insulin action, as well as improving mitochondrial health and function, particularly through the activation of AMP‐kinase (AMPK) (Ruegsegger et al., 2019; Wang et al., 2019). AMPK is a well‐known target for improving metabolic homeostasis; activation of AMPK in the liver reduces glucose production (Rena et al., 2017), while in skeletal muscle AMPK increases glucose uptake through non‐Akt dependent GLUT4 translocation, and improves insulin action (Jeon, 2016; Kim et al., 2016). The targeting of AMPK with the use of MET has been shown to be effective in peripheral IR, but whether these effects persist with brain IR are not yet known.

It is possible that the effects of IR on brain metabolism and synaptic function might be an underlying cause of the cognitive impairment observed with AD development. To this end, targeting the improvement of brain IR through the activation of AMPK using MET, as a possible treatment for AD is enticing. However, whether targeting AMPK activation will improve insulin signaling and synaptic health in the brain is unclear. Moreover, it is possible that AMPK activation itself may be detrimental to synaptic health; post‐mortem AD brains exhibit substantial AMPK hyper‐phosphorylation, of which the impact on AD progression or pathogenesis remains unexplained (Vingtdeux et al., 2011). Our group and others have shown that in vitro AMPK activation impairs synaptic protein content and neuronal morphology, inducing synapse loss through mTORC1 inhibition (Domise et al., 2019; Yang et al., 2022). Likewise, Potter et al. (2010) have shown that activation of AMPK impairs synaptic function in ex‐vivo brain slices through repressing mTORC1, and that these effects were corrected or improved with AMPK inhibition, as were synaptic impairments induced by A*β* (Ma, Chen, et al., 2014). These studies were not conducted under conditions of peripheral or brain IR meaning that it is unknown whether results would be similar under conditions of metabolic dysregulation. It is also unclear whether MET will produce similar neuronal impairments through AMPK activation, nor whether the treatment of IR with MET will recover IR‐driven synaptic impairments.

This study had two specific aims to address AMPK signaling under conditions of metabolic dysregulation. First, we examined whether IR and MET distinctly impact synaptic protein content and morphology in neurons through separate or coordinated mechanisms; and second, we examined whether IR‐induced impairments are improved with the addition of MET.

## 2 METHODS AND MATERIALS

### 2.1 Cell line and treatments

SH‐SY5Y Human Neuroblastoma cells were donated internally from Brock University or purchased from ATCC131 (cat# CRL‐2266; sex of cell line is female). Use of this cell line was approved by the Research Ethics Board at Brock University (#17–397). Cells were cultured using high glucose Dulbecco's Modified Eagles Media (DMEM Sigma D6429), containing 10%, 1%, 0.1% (v/v) FBS (Sigma F1051) for seeding, differentiation, and treatments respectively, as well as 5% non‐essential amino acid (Sigma M7145), and 1% Penicillin/Streptomycin (Sigma P4333). Unless otherwise stated, cells were seeded at 150,000 cells/mL on 12‐well culture plates (VWR 10062–894) in triplicate. and cultured until ~90%–95% confluency before differentiation. Differentiation was accomplished with retinoic acid (RA; 1 μg/mL; Milipore Sigma R2625) for 5 days. Differentiated cells were then assigned to one of the following 4 treatments: (1) Control media (H_2_O vehicle; Ctl); (2) High‐insulin (100 nM; HI; Eli Lilly 00586714); (3) Metformin (2 or 5 mM; MET; abcam ab120847); (4) High‐insulin + Metformin (100 nM HI+2 mM Met). To induce IR, cells were incubated with 100 nM insulin (INS) for 24 h following 5 days of differentiation with RA. This method of high‐insulin‐induced IR has previously been demonstrated in SH‐SY5Y cells and other cell types (Amine et al., 2021; Mayer & Belsham, 2010; Tian et al., 2019; Vlavcheski et al., 2020). 2 mM Metformin was used to induce the phosphorylation of AMPK T172 (Benito‐Cuesta et al., 2021; Lu et al., 2016; Son et al., 2016). A Trypan blue exclusion assay was conducted for both HI and MET treatments over 24 h to determine cell viability with these treatments as described by Strober (2015). Differentiated cells were treated for a total of 48 h as follows: (1) Ctl: 24 h vehicle followed by 24 h Vehicle; (2) HI: 100 nM insulin (24 h HI followed by 24 h HI); (3) MET: 24 h vehicle followed by 24 h 2 mM metformin; (4) HI/MET: 24 h 100 nM insulin followed by 24 h 100 nM INS+2 mM MET. Cells were then collected for analysis via western blotting (WB), RT‐qPCR, or immunofluorescence (IF).

### 2.2 Insulin stimulation

To examine the effectiveness of the high‐insulin‐induced IR, an acute insulin stimulation experiment was performed. This assessment of insulin responsiveness was adapted from and conducted as previously described (Amine et al., 2021; Marko et al., 2020). Briefly, cells were incubated in 0.1% (v/v) FBS media containing either vehicle control (dH_2_O) or 100 nM INS overnight, after which time fresh 0.1% FBS media containing 100 nM INS was added acutely for 30 min. Cells were then collected and analyzed by WB to assess Akt T308 and S473 phosphorylation status, as well as mTOR subunit S2448 phosphorylation status. Impaired insulin signaling was indicated by a decreased ability of the acutely added INS to increase Akt and mTOR phosphorylation.

### 2.3 Western blotting

Cells were lysed in 200 μL cell lysis buffer (NP40 Cell Lysis Buffer (Life Technologies; CAT# FNN0021) supplemented with 34 μL phenylmethylsulfonyl fluoride and 50 μL protease inhibitor cocktail (Sigma; CAT# 7626‐5G, CAT# P274‐1BIL). Cell lysates were then sonicated (10 second pulses, 2 pulses per sample; Fischer Scientific Sonic Dismembator 100). A bicinchoninic acid assay was performed to determine the protein concentration of the lysates. Samples were prepared (1 μg/μL) and equal amounts of protein were then electrophoretically separated on 10% SDS‐PAGE gels and transferred to nitrocellulose membranes (GE Life Science Ca. 10600002, 0.45 μm). Membranes were blocked for 90 min at room temperature in 5% non‐fat dry milk‐TBST (tris‐buffered saline/0.1% tween 20). Membranes were then incubated in primary antibody diluted 1:1000 in 5% BSA (Bovine Serum Albumin)‐TBST overnight at 4°C with gentle agitation. The following day, membranes were incubated for 1 h at room temperature with the appropriate secondary antibodies (1:2000; Donkey anti‐rabbit IgG (H+L), #711–035‐152, Goat anti‐mouse IgG (H+L), #115–035‐003 Jackson Immunoresearch) in 1% BSA‐TBST. Membranes were rinsed 3× 5 min in TBST and proteins were visualized by western Lightning Plus‐ECL (Perkinelmer NEL103E001EA) using a ChemiDoc Imaging System (Bio‐Rad).

Band densitometry was quantified using Alpha Innotech software (Santa Clara, CA). Total and phosphorylated AMPK levels were measured using specific primary antibodies (Cell Signaling #2531 (Koh et al., 2022); Cell Signaling #2535 (Bartolacci et al., 2022)—recognizes phosphorylated Threonine 172). Total raptor (Cell Signaling #2280 (Sanders et al., 2022)) and phosphorylated raptor (S792, Cell Signaling #2083 (Sanders et al., 2022)) levels were also measured alongside total P70S6K (Cell Signaling #9202 (Zhang et al., 2021)), phosphorylated P70S6K (Santa Cruz SC‐11759 (Xiao et al., 2002)), total ULK (Cell signaling #8054 (Wu et al., 2020)), and ULK S555 (cell signaling #5869 (Wu et al., 2020)). Synaptophysin (Cell Signaling #5461 (Wu et al., 2018)), NeuN (Cell Signaling #24307 (Tang et al., 2021)), PSD‐95 (post‐synaptic marker, Cell Signaling #36233 (Shui et al., 2022)), Homer‐1 (post‐synaptic marker, SC‐136358 (Wang et al., 2014)), SNAP‐25 (Cell signaling #5308 (Polishchuk et al., 2023)), VAMP2 (Cell signaling #13508 (Arrojo et al., 2019)) were measured as synaptic and neuronal markers. Total IR*α* (Cell signaling #74118 (Dall'Agnese et al., 2022)), total IR*β* (Cell signaling #23413 (Dall'Agnese et al., 2022)), IRS1 S636 (Cell signaling #2388 (Uddin et al., 2019)), total IRS1 (Cell signaling #2390 (Tang et al., 2019)), mTOR S2448 (Cell signaling #2971 (Luo et al., 2022)), total mTOR (Cell signaling #2972 (Luo et al., 2022)), Akt T308 (Cell signaling #9275 (Mathieu et al., 2019)), Akt S473 (Cell signaling #4058 (Marko et al., 2020)), and total Akt (Cell signaling #4685 (Marko et al., 2020)) were measured as markers of insulin signaling. Ponceau S, *α*‐tubulin (Cell signaling #2144 (Mertins et al., 2021)), GAPDH (Abcam ab8245 (Luo et al., 2023)) or *β*‐Actin (Abcam ab8227 (Moll et al., 2023)) were utilized as loading controls.

### 2.4 RT‐qPCR

Changes in mRNA expression were determined using real‐time quantitative PCR as described previously (Baranowski et al., 2021; MacPherson et al., 2015; Marko et al., 2022). RNA was isolated from SH‐SY5Y cells seeded, differentiated, and treated as described above. A RNeasy kit was used according to the manufacturer's instructions (Qiagen 74106). Following RNA isolation, a DNAse‐free treatment was used according to the manufacturer's instructions (AM1906, Thermo) to purify the samples (remove genomic DNA) before use. Each sample concentration and purity of the isolated mRNA was determined using the NanoVue plus Nano‐drop system (GE healthcare). mRNA samples were prepared at 1 ng/mL using RNase‐free water. mRNA was extracted and reversed transcribed into cDNA. Complementary DNA strands were created with primers and dNTPs at a 1:1 ratio, as well as a master mix containing 5× FSB, DTT, RNase out, and SuperScript II Reverse Transcriptase. Each sample was loaded in duplicate and contained 10 mL of PCR master mix, 4 mL of RNase‐free water, 1 mL of gene expression assay, and 5 mL of cDNA, which was diluted with 80 mL of RNase‐free water. Gene expression was measured for *DLG4* (Thermo Fischer Hs00176354_m1) and *HOMER1* (Thermo Fischer Hs00188676_m1) as well as *GAPDH* (thermos Fischer Hs02786624_m1), which was used as a reference gene. Relative differences in mRNA expression were determined using the 2^−∆∆CT^ method (Livak & Schmittgen, 2001).

### 2.5 Immunofluorescence

SH‐SY5Y cells were seeded at 75,000 cells/mL, differentiated, and grown on MatTek glass bottom culture dishes until they reached 75% confluency (35 mm Dish, No. 1.5 Coverslip, 10 mm Glass Diameter, Collagen Coated). Cells were then fixed using 4% paraformaldehyde and permeabilized using 20% triton X‐100 before being incubated in a buffer containing phalloidin actin stain (Abcam; ab176757 (Wang et al., 2022)) and DAPI stain (Abcam; ab228549). Images were captured using a Biotek Cytation5 cell imaging reader. Immunofluorescent (IF) images were captured in a 2 × 2 image grid (10×) where DAPI was used to determine cell number and phalloidin stain was used to determine cell size and neurite projection length. Neuron size (cell diameter) & area (total cell size), and length (projection length) & area (total projection size) of neurites were analyzed using the Biotek Gen5 imaging software. Neuron size and area were defined by fluorescent signals measured 1–4 μm from the nucleus defined by DAPI stain, and neurite length and area were defined by fluorescent signals measured 4–40 μm from the nucleus defined by the actin stain. All stitched montage images were preprocessed for background flattening (rolling ball diameter 628 um, 210 pixels) on all channels before cellular analysis.

### 2.6 Statistical analysis

Differences in protein content/phosphorylation status, cell size, neurite length, and fluorescent levels were determined using a one‐way ANOVA followed by a Fischer's LSD *post hoc* test. Differences in the acute insulin stimulation treatments were determined using a two‐way ANOVA followed by a Fischer's LSD *post hoc* test. Differences in cell viability were determined using a two‐tailed student's *t*‐test. A value of *p* < 0.05 was considered significant. All data are reported as mean ± SD of 3–7 independent experiments (*N*) where one *N* represents 3 pooled replicates. Pooled replicates were taken from identical treatments from within the same plate.

## 3 RESULTS

### 3.1 Experimental model of insulin resistance and metformin treatments

**FIGURE 1:**
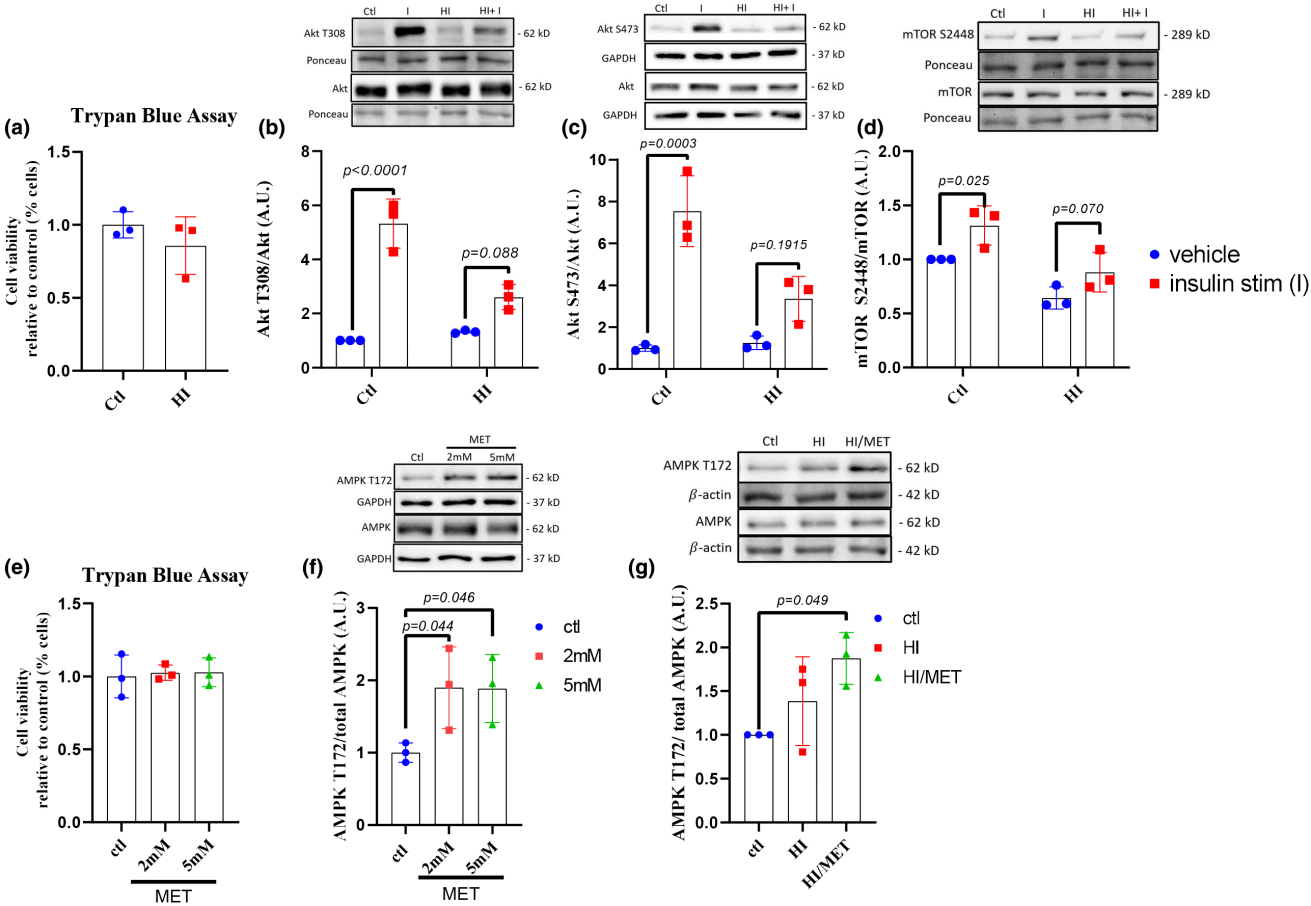
IR experimental design and MET dose‐response. (a) Trypan Blue cell viability assay following 24 h insulin treatment (100 nM) (HI). (b) Akt T308 phosphorylation following 24 h HI treatment (100 nM) and acute stimulation with insulin (I; 100 nM). (c) Akt S473 phosphorylation following 24 h HI treatment (100 nM) and acute stimulation with insulin (I; 100 nM). (d) mTOR S2448 phosphorylation following 24 h HI treatment and acute stimulation with insulin (I; 100 nM). (e) Trypan Blue cell viability assay following 24 h metformin (MET) treatment (2 mM & 5 mM). (f) AMPK T172 phosphorylation following MET dose‐response (2 mM & 5 mM). (g) AMPK T172 phosphorylation following 48 h HI treatment (100 nM) or 24 h HI (100 nM) followed by 24 h HI (100 nM)+MET (2 mM). Percent change represented as mean ± SD, significance (*p* < 0.05) represented by symbols: *p < 0.05, **p < 0.01, ***p < 0.001, ****p < 0.0001 indicates the difference between groups as determined using a student *t*‐test (a), one‐way (e–g) or two‐way (b–d, h) ANOVA followed by Fischer's LSD post hoc analysis.

The development of our experimental model took place over 2 phases, with the first being the development of the insulin‐resistant model and the second being the optimization of the concentration of metformin required to induce AMPK activation. To induce insulin resistance, cells were treated with 100 nM insulin for 24 h (HI). This dose has been used previously by others to disrupt insulin signaling (Amine et al., 2021; Huang et al., 2002; Mayer & Belsham, 2010; Pederson et al., 2001; Sun et al., 1999; Tian et al., 2019; Vlavcheski et al., 2020), however, its use in SH‐SY5Y cells has not been reported prior to this study (Amine et al., 2021). As such, cell viability was assessed with a Trypan Blue Exclusion Assay, which determines whether cell membranes remain intact and can actively exclude trypan blue; dead cells that do not possess intact membranes will thus be stained by trypan blue (Strober, 2015). Following the treatment of cells with 100 nM insulin for 24 h, trypan blue exclusion showed comparable cell viability to controls (Figure 1a; student *t*‐test, *p* = 0.3415). Acute insulin responsiveness was then assessed by exposure to fresh media with or without 100 nM insulin for 30 min, followed by WB analysis of the insulin signaling markers, Akt T308 and S473 phosphorylation. Impairment of increases in the phosphorylation of both Akt phosphorylation sites are commonly used markers indicative of impaired insulin signaling. Two‐way ANOVAs indicated an interaction between phosphorylation status and pre‐exposure to insulin for both Akt T308 (two‐way ANOVA, interaction *p* = 0.0009, *post hoc*_ctl‐I_ *p* < 0.0001, *post hoc*_HI‐HI+I_ *p* = 0.0884) and Akt S473 (two‐way ANOVA, interaction *p* = 0.0054, *post hoc*_ctl‐I_ *p* = 0.0003, *post hoc*_HI‐HI+I_ *p* = 0.1915). Phosphorylation status of Akt T308 and S473 was increased with a 30‐min insulin stimulation in control cells treated with 0.1% media alone, but this increase was prevented following a 24 h pre‐treatment with 100 nM insulin (Figure 1b,c). This reduced Akt signaling in the HI group was also reflected in the lack of mTOR S2448 phosphorylation (two‐way ANOVA: main effect for treatment *p* = 0.0012, *post hoc*_ctl‐I_ *p* = 0.025, *post hoc*_HI‐HI+I_ *p* = 0.0695) (Figure 1d), indicating that cells pre‐treated with 100 nM insulin have impaired insulin signaling, indicative of the development of IR.

To establish the effective dose of MET, we exposed cells to either 2 mM or 5 mM MET, doses used in previous studies (Benito‐Cuesta et al., 2021; Lu et al., 2016; Son et al., 2016), for 24 h. Cells exposed to 2 mM or 5 mM MET showed no reduction in viability compared to the vehicle‐treated control cells, as determined by the Trypan Blue Exclusion assay (Figure 1e; one‐way ANOVA *p* = 0.9382) and both doses showed equivalent increases in AMPK T172 phosphorylation status (Figure 1f; one‐way ANOVA *p* = 0.0142, *post hoc*_ctl‐2mM_ *p* = 0.0153, *post hoc*_ctl‐5mM_ *p* = 0.007, *post hoc*_2mM–5mM_ *p* = 0.2529). As such, the 2 mM dose was selected for use in subsequent experiments.

We next investigated whether HI treatment would impact AMPK phosphorylation and whether MET would still be effective at increased AMPK phosphorylation. Cells were incubated with 100 nM insulin for 24 h followed by 24 h 100 nM insulin (HI) or 100 nM insulin for 24 h followed by both 100 nM insulin and MET (HI/MET). AMPK T172 phosphorylation status was higher following HI/MET treatment (Figure 1g: one‐way ANOVA *p* = 0.0137, *post hoc*_ctl‐HI/MET_ *p* = 0.0495) compared to both control and HI.

### 3.2 Impact of insulin and metformin on insulin signaling

**FIGURE 2:**
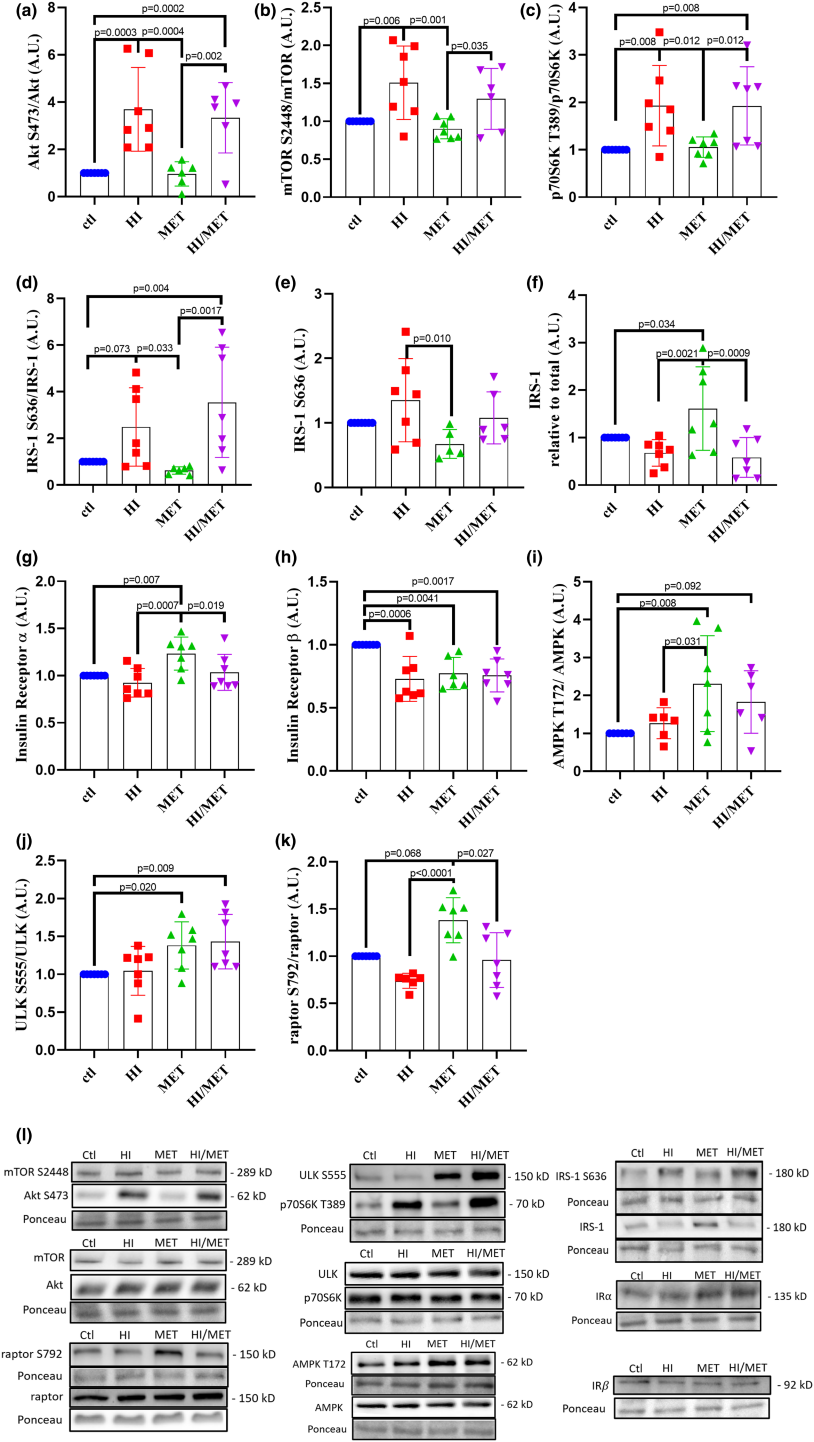
Signaling changes following 48 h of HI, MET, or HI/MET treatments. (a) AKT S473 phosphorylation, (b) mTOR S2448 phosphorylation, (c) p70S6K T389 phosphorylation, (d) IRS‐1 S636 phosphorylation, (e) Total IRS‐1 content, (f) total IR*α* content, (g) total IR*β* content, (h) AMPK T172 phosphorylation, (i) ULK S555 phosphorylation, (j) raptor S792 phosphorylation, as a marker of mTORC1 inhibition. Percent change is represented as mean ± SD. *p < 0.05, **p < 0.01, ***p < 0.001, ****p < 0.0001 and indicate differences between groups as determined using a one‐way ANOVA followed by Fischer's LSD *post hoc* analysis.

MET is the most prescribed T2D drug that is used to improve the insulin signaling and glycemic control which is commonly dysregulated in metabolic disease (Kim et al., 2016; Rena et al., 2017). MET is highly effective in improving metabolic dysregulation in the periphery, but its effectiveness in improving neuronal insulin signaling is unknown. As such, we investigated the ability of MET to improve canonical insulin signaling of IR cells. Following 48 h of HI, MET, or HI/MET treatment, Akt S473 (one‐way ANOVA *p* = 0.0002) and mTOR S2448 (one‐way ANOVA *p* = 0.0062) phosphorylation status was higher in both HI (Akt *post hoc*_ctl‐HI_ = 0.0003; mTOR *post hoc*_ctl‐HI_ = 0.0019) and HI/MET (Akt *post hoc*_ctl‐HI/MET_ = 0.0019; mTOR *post hoc*_met‐HI/MET_ = 0.0353) groups compared to Ctl and MET treatments (Figure 2a,b). This higher mTOR S2448 phosphorylation was accompanied by higher p70s6k T389 phosphorylation, indicative of greater mTORC1 activity (Figure 2c; one‐way ANOVA *p* = 0.0063; *post hoc*_ctl‐HI_ = 0.008; *post hoc*_ctl‐HI/MET_ = 0.0082). IRS‐1 S636 phosphorylation status was next investigated, as IRS‐1 S636 is a target of mTORC1 through the activation of p70s6k (Copps & White, 2012). Increased phosphorylation of this IRS‐1 residue as a result of increased mTORC1 activity (Copps & White, 2012; Ozes et al., 2001) has been directly associated with brain IR and cognitive impairment (Talbot et al., 2012).

The ratio of IRS‐1 S636/IRS‐1 approached significance (one‐way ANOVA *p* = 0.0051, *post hoc*_ctl‐HI_ *p* = 0.073) following HI treatment and was higher following HI/MET treatment relative to ctl (*p* = 0.0039) and MET (*p* = 0.0017) groups (Figure 2d). While it approached significance (Figure 2e; one‐way ANOVA *p* = 0.0692), no significant increase in IRS‐1 S636 phosphorylation was seen in the HI or HI/MET groups relative to control (*post hoc*_ctl‐HI_ *p* = 0.1213, *post hoc*_ctl‐HI/MET_ *p* = 0.7355); only HI was seen to increase relative to MET (*post hoc*_HI‐MET_ *p* = 0.0099). Total IRS‐1 content, however, was found to be higher in the MET group relative to all other groups (Figure 2f; one‐way ANOVA *p* = 0.0038, *post hoc*_ctl‐MET_ *p* = 0.0337, *post hoc*_HI‐MET_ *p* = 0.0021, *post hoc*_MET‐HI/MET_ *p* = 0.0009). Further, insulin receptor‐*α* (IR*α*) and insulin receptor‐*β* (IR*β*) were examined as both IR*α* and IR*β* constitute the two major subunits of the insulin receptor that bind insulin to induce downstream insulin action, and reductions in their expression have been associated with the development of IR. IR*α* receptor levels were higher with MET treatment compared to Ctl (one‐way ANOVA *p* = 0.0053, *post hoc*_ctl‐MET_ *p* = 0.0074) (Figure 2g) in a similar manner to total IRS‐1, whereas IR*β* levels were lower in all groups relative to control (Figure 2h; (one‐way ANOVA *p* = 0.0022, *post hoc*_ctl‐HI_ *p* = 0.0006. *post hoc*_ctl‐MET_ *p* = 0.0041, *post hoc*_ctl‐HI/MET_ *p* = 0.0017)).

### 3.3 Impact of insulin and metformin on AMPK and mTORC1 signaling

mTORC1, a major marker of cell growth and proliferation, is a target of both AMPK and Akt, which inhibit or induce mTORC1‐driven protein synthesis/cell growth, respectively. As our previous work showed that AMPK activation and downstream signaling through mTORC1 plays a role in synaptic protein expression of retinoic acid‐differentiated SH‐SY5Y cells (Yang et al., 2022), we next sought to examine the impact of MET, and the effect of treating IR with MET, on the regulation of these pathways.

MET alone showed a higher AMPK phosphorylation status when compared to the Ctl‐ and HI‐treated groups (Figure 2i; one‐way ANOVA *p* = 0.038, *post hoc*_ctl‐MET_ *p* = 0.0085, *post hoc*_HI‐MET_ *p* = 0.0312). This increase was also observed in ULK S555, the direct downstream target of AMPK when compared to both Ctl and HI groups (Figure 2i; one‐way ANOVA *p* = 0.0156, *post hoc*_ctl‐MET_ *p* = 0.0203, *post hoc*_HI‐MET_ *p* = 0.0388). Despite the HI/MET treatment not showing higher AMPK T172 phosphorylation (post = hoc_ctl‐HI/MET_ *p* = 0.0924), HI/MET treatment did show higher ULK S555 phosphorylation (Figure 2j; post = hoc_ctl‐HI/MET_ *p* = 0.0097), suggesting that treatment of INS with MET induces the activation of ULK. This would be despite AMPK T172 phosphorylation being no different to control (Figure 2i) and despite no increase in the AMPK‐specific inhibitory phosphorylation of raptor S792 subunit of mTORC1 (Figure 2k), implying that AMPK signaling is not active with HI/MET treatment.

The formation of mTORC1 is inhibited through the phosphorylation of its raptor subunit at the S792 phosphorylation site and mTORC1 activity can be assessed through the increased phosphorylation of the mTORC1 downstream target, p70s6k. Comparison of these 2 phosphorylation targets can therefore be used as an indirect measure of mTORC1 activity, related to cellular energy status/protein synthesis. We found that the MET treatment group had higher phosphorylation of the AMPK‐specific site of raptor S792 (Figure 2k; one‐way ANOVA *p* < 0.0001, *post hoc*_ctl‐MET_ *p* = 0.0068), with no change in p70s6k phosphorylation (Figure 2c) compared to controls, indicating an impairment of mTORC1 signaling. Similarly, both HI and HI/MET groups showed higher p70s6k phosphorylation compared to MET and control groups indicating that with HI and HI/MET treatments, mTORC1 signaling is active (Figure 2b).

### 3.4 Neuronal and synaptic modification

**FIGURE 3:**
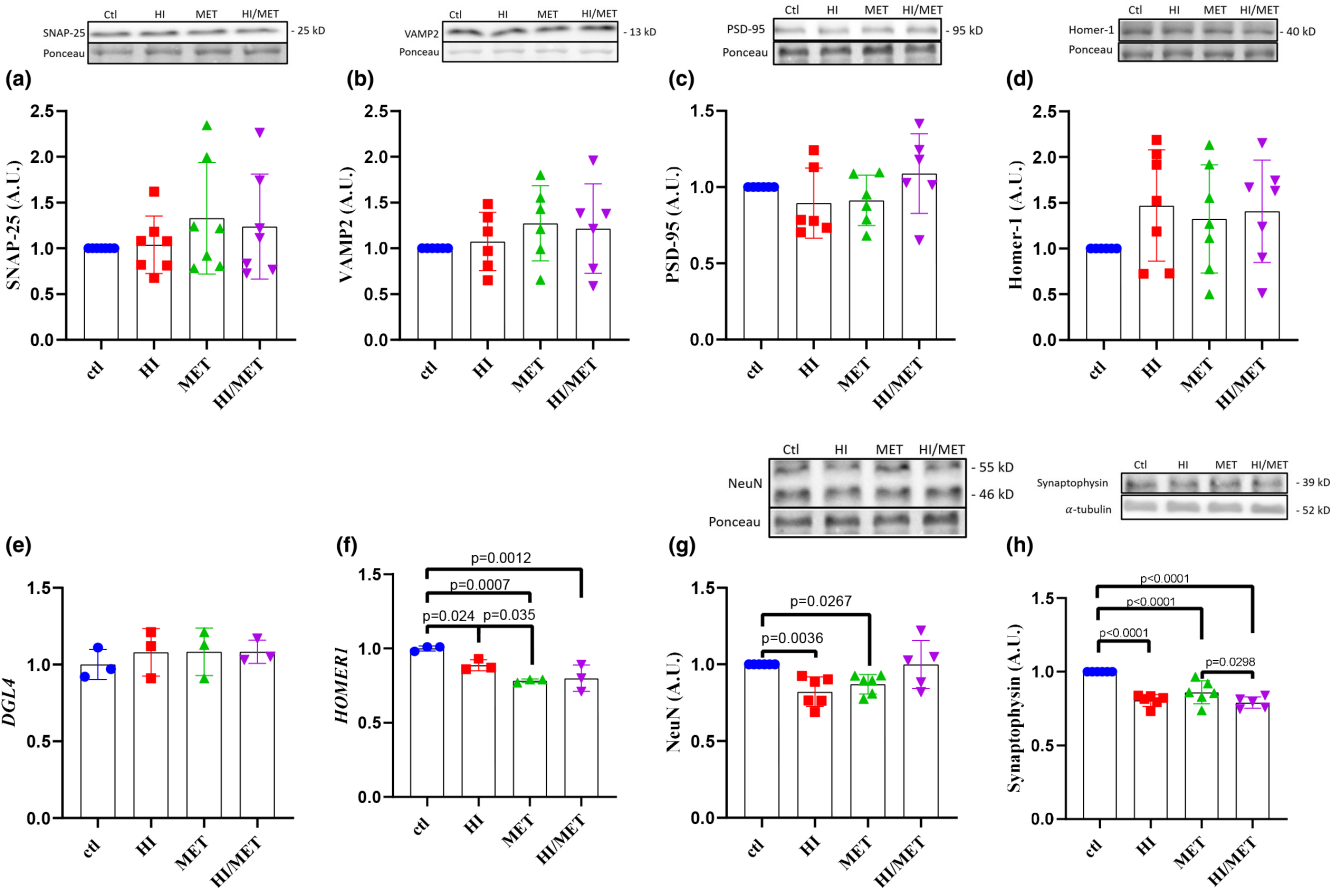
Regulation of key synaptic markers following HI, MET, or HI/MET treatment. (a) total SNAP‐25 protein content, (b) total VAMP2 protein content, (c) total PSD‐95 protein content, (d) total Homer‐1 protein content, (g) DLG4 mRNA content, (f) HOMER1 mRNA content, (g) total NeuN protein content, and (h) total Synaptophysin protein content. Percent change represented as mean ± SD; *p < 0.05, **p < 0.01, ***p < 0.001, ****p < 0.0001 indicate differences between groups as determined using a one‐way ANOVA followed by Fischer's LSD *post hoc* analysis.

Next, we investigated whether IR impacts neuronal markers associated with synaptic function and whether treatment with MET would improve any proteome‐level impairments caused by IR. Secondly, we examined whether MET‐induced activation of AMPK would induce similar reductions in neuronal protein content to those we have previously shown with direct AMPK activation (Yang et al., 2022). We chose to investigate major protein markers that are required for synaptic transmission; SNAP‐25 and VAMP2 are presynaptic proteins associated with neurotransmitter vesicle release (Risselada et al., 2011); PSD‐95 and Homer‐1 are post‐synaptic density markers associated with neurotransmitter receptor stabilization (Ma, Paul, et al., 2014; Shiraishi‐Yamaguchi & Furuichi, 2007; Taft & Turrigiano, 2014). Reductions in these presynaptic and postsynaptic markers have been associated with cognitive impairment and AD progression (Berchtold et al., 2013, 2019; Luo et al., 2012). Following 48 h of HI, MET, or HI/MET treatments, no significant changes in presynaptic (SNAP‐25, one‐way ANOVA *p* = 0.4691; VAMP2, one‐way ANOVA *p* = 0.5351) or postsynaptic (PSD‐95, one‐way ANOVA *p* = 0.3082; Homer‐1, one‐way ANOVA *p* = 0.4010) protein content (Figure 3a–d) was seen.

As no changes in protein content were seen, we next investigated whether changes in mRNA content occurred. As the expression of both PSD‐95 and Homer‐1 are paramount for post‐synaptic density and synaptic function, we investigated whether the mRNA expression of these markers was affected by our treatments. We found no change in *DLG4* (mRNA encoding for PSD‐95; Figure 3e; one‐way ANOVA *p* = 0.8102) following any treatment. However, *HOMER1* mRNA content was significantly altered (one‐way ANOVA *p* = 0.0026, *post hoc*_ctl‐HI_ *p* = 0.0239, *post hoc*_ctl‐MET_ *p* = 0.0007, *post hoc*_ctl‐HI/MET_ *p* = 0.0012) and was reduced in all treatment groups relative to control (Figure 3f). Additionally, *HOMER1* mRNA was also significantly reduced following MET treatment compared to HI (Figure 3f; *p* = 0.035) indicating that MET activation of AMPK alone, seems to lower *HOMER1* mRNA content more than HI treatment.

As no changes in protein expression for pre or postsynaptic markers were seen, we next investigated whether changes in other major neuronal makers, known to be reduced with brain IR (Duarte et al., 2019; Sun, 2006) and AD (Mattson & Arumugam, 2018), were affected by our treatments. NeuN is a marker of mature neurons, and synaptophysin, is a ubiquitously expressed synaptic marker. Changes in NeuN were found (one‐way ANOVA *p* = 0.0069, *post hoc*_ctl‐HI_ *p* = 0.0036, *post hoc*_ctl‐MET_ *p* = 0.0267, *post hoc*_HI‐HI/MET_ *p* = 0.0052, *post hoc*_MET‐HI/MET_ *p* = 0.0341), with reductions in NeuN in both HI and MET groups, compared to Ctl and HI/MET treatments (Figure 3g). Synaptophysin levels were also changed (one‐way ANOVA *p* < 0.0001, *post hoc*_ctl‐HI_ *p* < 0.0001, *post hoc*_ctl‐MET_ *p* < 0.0001, *post hoc*_ctl‐HI/MET_ *p* < 0.0001, *post hoc*_MET‐HI/MET_ *p* = 0.0293) and were lower in all treatment groups compared to control, with HI/MET treatment showing greater reductions in protein content compared to MET alone (Figure 3h).

### 3.5 Cell morphology

As modifications in synaptic function are usually a result of changes in specific regions of neurons (axons and dendrites), changes in protein content of the whole neuron might not fully reflect adjustments in the synapses nor overall changes in cell function. As such, changes in neuron morphology were analyzed to provide additional information about neuronal and/or synaptic function. The focus of this morphological analysis was directed to changes in soma size (cell size, and total cell area), as well as neurites (neurite area, and neurite length), as they represent the two major regions that define neurons (Amato et al., 2011; Yang et al., 2022). Reduced soma size has been reported during impaired insulin signaling (knockout models of insulin‐like growth factor 1 receptor; IGF‐1R), which has been associated with impaired cognitive function (Gontier et al., 2015). Reductions in neurite length/area have also been associated with impaired synaptic transmission, as well as impaired neuronal health (Amato et al., 2011; Gontier et al., 2015).

**FIGURE 4:**
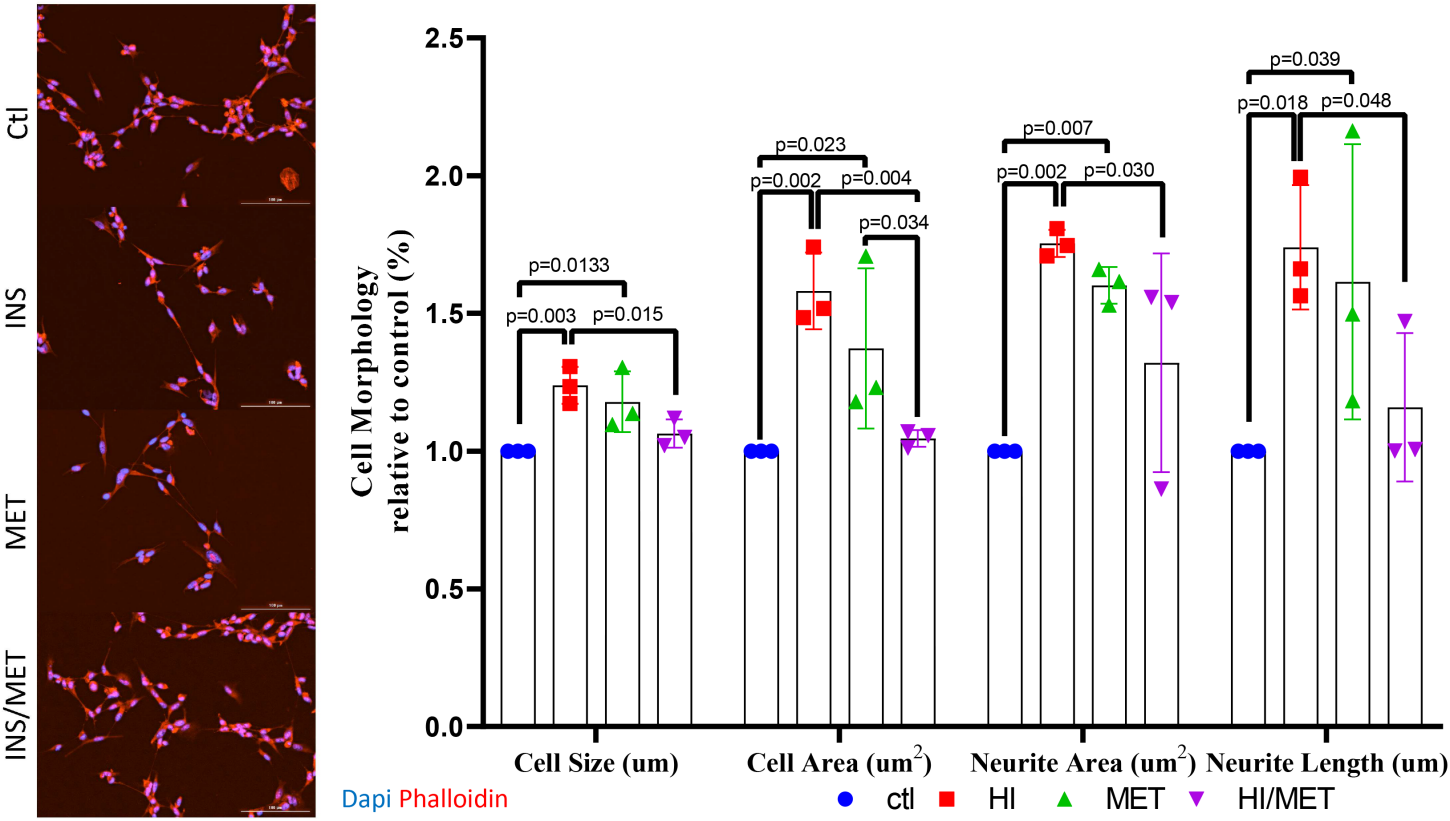
Examination of SH‐SY5Y cell size and neurite projections following 24 h HI, MET, or HI/MET treatment. Representative IF images for morphological changes in neuron size (cell diameter), neuron area (total cell size), neurite area (total neurite projection area), and neurite projection length. DAPI nucleus stain shown in blue, and Phalloidin cytoskeleton Actin stain shown in red. Results represent independent experiments (*n* = 3). All images were preprocessed for background flattening (rolling ball diameter 628 um, 210 pixels) on all channels before cellular analysis. Percent change represented as mean ± SD, *p < 0.05, **p < 0.01, ***p < 0.001, ****p < 0.0001 indicate differences between groups as determined using a one‐way ANOVA followed by Fischer's LSD *post hoc* analysis.

Cells treated with both HI and MET exhibited larger cell sizes (one‐way ANOVA *p* = 0.0111; *post hoc*_ctl‐HI_ *p* = 0.0029, *post hoc*_ctl‐MET_ *p* = 0.0133) and total cell area (one‐way ANOVA *p* = 0.0067; *post hoc*_ctl‐HI_ *p* = 0.0023, *post hoc*_ctl‐MET_ *p* = 0.0225) as well as greater neurite area (one‐way ANOVA *p* = 0.0084; *post hoc*_ctl‐HI_ *p* = 0.0019, *post hoc*_ctl‐MET_ *p* = 0.0066) and neurite length (one‐way ANOVA *p* = 0.050; *post hoc*_ctl‐HI_ *p* = 0.0180, *post hoc*_ctl‐MET_ *p* = 0.0391), compared to controls, however, these increases in cell size were absent with the combined HI/MET treatment (Figure 4; cell size *post hoc*_ctl‐INS/MET_ *p* = 0.2948, total cell area *post hoc*_ctl‐INS/MET_ *p* = 0.7357, neurite area *post hoc*_ctl‐INS/MET_ *p* = 0.0888, neurite length *post hoc*_ctl‐INS/MET_ *p* = 0.5413). Larger neurite and cell size are commonly associated with greater cell growth and protein synthesis in support of synaptic transmission (Arendt, 2020; Fornasiero et al., 2010; Prem et al., 2020; Reese & Drapeau, 1998), suggesting that greater cell/neurite growth following HI and MET treatments might support greater synaptic function; increases that are ablated when HI cells are treated with MET (HI/MET).

## 4 DISCUSSION

Our understanding of how metabolic dysregulation, like IR and T2D, can impact brain metabolism and influence synaptic impairment is poorly understood. This is in spite of research demonstrating that the development of IR and metabolic dysregulation are known risk factors for AD, and are highly associated/correlated with its development (Norton et al., 2014) and progression (Talbot, 2014; Velazquez et al., 2017). Due to the strong relationship between IR, T2D, and AD, the re‐purposing of effective medications used to treat peripheral metabolic dysregulation is being explored as treatments for AD. One noteworthy drug, MET, has garnered substantial interest for AD treatment owing to its efficacy in treating T2D. Despite some evidence to suggest that MET can improve the metabolic profile in the brain (Ruegsegger et al., 2019), whether MET will improve or impact synaptic health is unknown. It is also unknown whether the treatment of brain IR with MET will be beneficial for synaptic health. Thus, this study sought to examine the effects of IR on neuron morphology and synaptic health and to determine whether MET is effective in treating IR‐driven impairments in neuronal function. We determined that high‐insulin‐induced IR in SH‐SY5Y cells reduced the levels of two important neuronal proteins, NeuN and synaptophysin, and reduced mRNA levels of a major post‐synaptic protein, Homer‐1. When IR cells were treated with MET (HI/MET treatment), no improvement in the hyperphosphorylation status of any marker related to insulin signaling was observed. The HI/MET treatment recovered NeuN content, but significantly reduced synaptophysin content compared to MET treatment alone. Additionally, both HI and MET treatments alone increased neuron size/area and neurite size/length, but these increases were not present when IR neurons were treated with MET (HI/MET).

### 4.1 Insulin resistance alters neuron morphology and synaptic proteins

Our understanding of insulin signaling is not as well studied in the brain as it is in the periphery. While there is evidence outlining a role for insulin action in brain (Benedict et al., 2004; Lee et al., 2005, 2011), these studies have only ever been conducted with either supraphysiological concentrations of insulin or under non‐pathologic conditions. As a result, very little is known about insulin signaling in brain pathologies, especially when discussing synaptic integrity in terms of neuronal protein content and neurite morphology.

**FIGURE 5:**
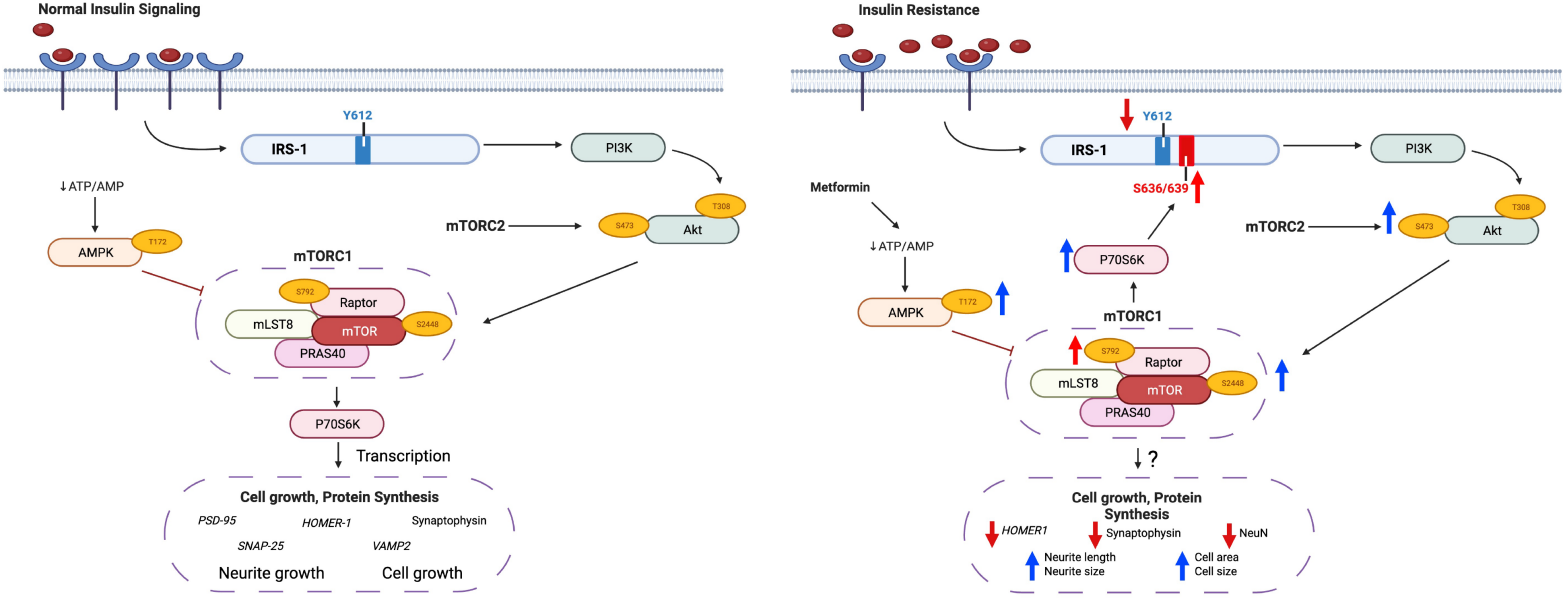
Proposed mechanisms for Insulin and Metformin signaling in neuron. *LEFT* Canonical insulin and AMPK signaling in response to physiological insulin concentrations and changes in energy status ([ATP] vs. [AMP]) respectively. With the binding of insulin to its receptor, phosphorylation of IRS‐1 tyrosine residues leads to the activation of Akt (T308 and S473), followed by the activation of mTORC1 (mTOR S2448) and p70s6k, which leads to cell growth and protein synthesis via transcription. In neurons, mTORC1 activation leads to synaptic protein synthesis, neurite outgrowth, and cell proliferation. Alterations in ATP/AMP concentrations, leading to greater AMP, induce the phosphorylation and activation of AMPK (T172). AMPK activation induces the inhibitory phosphorylation of the raptor subunit (S792) of mTORC1 leading to the inhibition of mTORC1, preventing synaptic protein synthesis, neurite outgrowth, and cell proliferation. *RIGHT* Disruptive insulin signaling (insulin resistance) in response to pathological insulin concentrations (hyperinsulinemia) and the impact of metformin on AMPK signaling. RED indicates inhibitory phosphorylation; BLUE indicates promotive phosphorylation. Prolonged, high concentrations of insulin are associated with reduced insulin receptor content, a blunted Akt response (Akt T308 and Akt S473), chronically elevated mTORC1 activation (mTOR S2448 phosphorylation) inducing the hyperactivation of p70S6k, and the hyperphosphorylation of inhibitory serine residues of IRS‐1. Metformin indirectly lowers ATP/AMP concentrations leading to the activation of AMPK and inducing the inhibition of mTORC1 activity (through the inhibitory phosphorylation of raptor S792). Both metformin and insulin resistance lead to reductions in neuronal markers as well as increases in neurite length & area, and cell size & area.

Insulin signaling in the brain is initiated by binding of insulin to the insulin receptor. This leads to the recruitment and activation of insulin receptor substrate (IRS), the downstream activation of PI3K, increasing the phosphorylation and activation of Akt and leading to eventual mTORC1 activation (Figure 5 Left). Impairments in Akt action are observed with brain IR and AD, and Akt signaling impairments in the brain play a comparative role in dysregulated insulin action with that seen with T2D and peripheral IR (Huang et al., 2018). As it pertains to the brain, important observations by researchers such as Talbot et al. (2012) have shown that a reduction of insulin receptor content alongside greater inhibitory phosphorylation of IRS‐1 S636 is evident with both IR and AD post‐mortem brains (Moloney et al., 2010; Tramutola et al., 2015, 2020), pointing to other major markers in insulin signaling that are impacted with IR. In fact, it is likely that this inhibitory phosphorylation of IRS‐1 drives the dysregulation of insulin signaling in the brain (Copps & White, 2012; Zhang et al., 2008), and the hyperactivation of both mTORC1 and Akt seems to drive this IRS‐1 inhibition (Copps & White, 2012; Tramutola et al., 2015; Zhang et al., 2008). To this end, our data corroborate these previous findings involving pathological changes in insulin signaling in both AD and brain IR. We demonstrate that high concentrations of insulin exposure, as is commonly observed with T2D and IR, lead to reduced insulin receptor content, increased Akt phosphorylation, and hyperactive mTORC1 activation, all of which seem to directly lead to greater inhibitory IRS‐1 phosphorylation.

Alongside changes in synaptic protein content, an important aspect of examining synapses involves quantification of changes in neuronal morphology. Reductions in synapse number and neurite length/size have commonly been reported to occur within AD (DeKosky & Scheff, 1990; Scheff et al., 1990; Vingtdeux et al., 2011) and this synaptic loss has been suggested to directly contribute to the development of cognitive impairment observed with AD progression (Ferreira‐Vieira et al., 2016; Kashyap et al., 2019; Terry et al., 1991). While insulin has been shown to induce dendritic and synaptic growth in isolated neurons (Lee et al., 2011) and to increase the expression of key synaptic proteins (Lee et al., 2005), studies examining insulin action in the brain and on synaptic function are often conducted with physiological levels of insulin or with exogenous treatment that does not induce IR. To this end, our study is one of the first to outline changes in neuron/neurite morphology with high‐insulin‐induced IR; HI treatment induced an enlargement of both cell size/area as well as increased neurite area and length that are novel observations with respect to IR. However, while neurite enlargements might be indicative of enhanced synaptic contact under physiologically normal conditions, it is difficult to associate changes in size with increased synaptic contact. Neurite and cell enlargements have been previously observed in AD patients where brain biopsies and post‐mortem AD brains show an increase in neurite length and size despite a severe reduction in synaptic density (DeKosky & Scheff, 1990). These authors suggested that, when paired with cognitive data, these neurite enlargements seen with AD brains suggest a compensatory increase in size to maintain equivalent synaptic transmission rates despite a significant loss in the number of synaptic contacts (Bertoni‐Freddari et al., 1993; DeKosky & Scheff, 1990; Scheff et al., 1990). A second observation, that has also been reported in AD patients, suggests that abnormal neurite swelling likely occurs because of aberrant accumulation of axonal cargo and cytoskeletal proteins (Argueti‐Ostrovsky et al., 2021; Chassefeyre et al., 2021; Stokin et al., 2005) driven by defects in axonal transport and autophagic clearance mechanisms (Argueti‐Ostrovsky et al., 2021; Kobro‐Flatmoen et al., 2021). Given the association between metabolic dysregulation and intracellular impairments such as cellular transport and autophagy, it is likely that the neurite enlargements observed both by us and with previous AD reports implicate a strong metabolic component in the progression of cognitive decline. One limitation of our measures is the lack of functional synaptic outcomes that would definitively translate these observed neurite enlargements to impairments in synaptic function in a similar capacity to what has been observed in post‐mortem AD brains (Bertoni‐Freddari et al., 1993; DeKosky & Scheff, 1990; Scheff et al., 1990). As such, future work should aim to determine whether the observed morphological changes in neurite length result in reduced synaptic function. Overall, these findings point to high‐insulin exposure being a driving component of the pathology observed in AD (Talbot et al., 2012). Furthermore, while the exact mechanism remains unclear, our findings indicate prolonged, high levels of insulin exposure as an important factor for impairments in synaptic protein expression and potentially aberrant neurite swelling.

### 4.2 The impact of metformin‐driven AMPK activation on synapse regulation

Previous work by our group and others has shown that chronic activation of AMPK has a direct impact on regulating synaptic protein expression. Specifically, AMPK activation results in reductions in major markers of synaptic transmission (PSD‐95, Homer‐1, Snap‐25, GluN1, GluA1) (Domise et al., 2019; Yang et al., 2022), impairments in cell morphology (Yang et al., 2022), and impaired network activity (Domise et al., 2019). These results suggest that hyperactivation of AMPK may not be beneficial for brain health. However, these studies were only conducted when cell metabolism was unperturbed, meaning that the impact of AMPK activation on synaptic health when metabolic dysregulation is present, such as with IR, is unclear. Additionally, whether the activation of AMPK by MET is beneficial in treating synaptic defects driven by IR is also poorly understood and demands further investigation. MET has both AMPK‐dependent and AMPK‐independent effects (Rena et al., 2017; Townsend et al., 2021) and the activation of AMPK by MET has been shown to occur as a secondary result of the partial inhibition of electron transport OXPHOS complex I (Rena et al., 2017), therefore it is unknown whether the results of our previous work will be translatable, let alone what impact MET‐driven AMPK activity will have if it is used to treat IR‐driven metabolic dysregulation. Our results here now show that 24 h MET treatment reduced NeuN and synaptophysin content as well as reduced *HOMER1* mRNA. While NeuN content was not examined in our previous work, reductions in synaptophysin following MET treatment are in line with previous results shown by our group (Yang et al., 2022) as well as Domise et al. (2019) who demonstrated reductions in synaptophysin following chronic AMPK activation. In this study, we did not observe any reductions in pre‐ or post‐synaptic marker protein content; including the same markers previously shown to be reduced with chronic AMPK activation (Domise et al., 2019; Yang et al., 2022). However, in both our previous work and that of Domise et al, the minimum time at which impairments in protein content were observed, was 5 days (Yang et al., 2022) and 72 h (Domise et al., 2019) respectively which is in stark contrast to the 24 h MET treatment conducted in this study. As AMPK regulates protein synthesis through the inhibition of mTORC1‐driven transcription (Kim et al., 2016), it is possible that the lack of changes in synaptic markers is a result of the fact that the length of treatment used in this study was simply not long enough for substantial changes in protein turnover to produce significant effects and that longer‐term cell culture is necessary to determine whether this is the case (Li et al., 2010). In support of this, reductions in mRNA content for the post‐synaptic marker, Homer‐1, were observed with MET treatment after 24 h, and previous work has shown reduced Homer‐1 protein content in neurons following chronic AMPK activation (Domise et al., 2019; Yang et al., 2022). Our results also show that treatment with MET induced an increase in neuron size/area as well as neurite length/area compared to controls. This is in direct contrast to our previous work (Yang et al., 2022) as well as others (Amato et al., 2011) demonstrating reductions in both neurite length, size, and polarization following AMPK activation. Given the known off‐target effects of MET, it is not unusual for differences in outcomes to be observed when comparing results between types of AMPK activators (MET vs. A‐769662 (Yang et al., 2022) vs. AICAR (Amato et al., 2011)). However, one explanation for these differences may be that MET‐induced enlargements in neurite size and length are a response to cellular stress and reductions in synaptic protein content similar to those observed with IR treatments (Argueti‐Ostrovsky et al., 2021; Chassefeyre et al., 2021; Stokin et al., 2005). This would be despite MET and IR clearly inducing the activation of separate pathways where the underlying driver might be related to a perturbed metabolic state. When coupled with previous findings (Domise et al., 2019; Yang et al., 2022), our current results highlight a distinct role for MET in impacting synaptic mRNA expression and neuronal protein expression through AMPK activation; results that may describe the impairments in synaptic function seen with MET through the inhibition of mTORC1, such as the impaired synaptic transmission (long‐term potentiation) observed by both Potter et al. (2010) and Ma, Chen, et al. (2014).

### 4.3 Metformin does not improve insulin resistance but prevents changes in cell morphology

We then examined whether the impairments as a direct result of insulin‐induced IR could be improved through MET treatment. Our treatment of IR with MET showed no improvement in any markers of the insulin signaling cascade: reductions in insulin receptor content, increased Akt phosphorylation, hyper‐mTORC1 activity, and greater inhibitory IRS‐1 phosphorylation were all comparable to treatments with HI alone. These increases persisted, despite showing an activation of the AMPK signaling cascade, and are likely due to uninhibited mTORC1 activity. In support of a model in which hyperactive mTORC1 drives synaptic impairments, increases in the ratio of IRS‐1 S636 phosphorylation to total and a lack of raptor S792 phosphorylation were similarly observed with HI/MET treatment that was not seen with MET. Despite significant differences in signaling and protein content changes in both HI and MET treatments, comparable reductions in synaptophysin protein and homer‐1 mRNA levels occurred with all treatments when compared to controls. Additionally, the reductions seen with HI treatments persisted with treatment of HI with MET (HI/MET), such as protein content ablations in total IRS‐1 and IR*α* seen with the HI/MET treatment when compared to MET alone. To this end, these results suggest that treatment of IR with MET in SH‐SY5Y neuroblastomas does not prevent IR‐driven impairments in synaptic protein content and that these impairments are likely driven by mTORC1. Future experiments employing rapamycin will be required to fully explore the extent of mTORC1 influence on mediating any synaptic impairments. Despite comparable reductions in other synaptic markers, HI/MET did not impair NeuN content compared to HI and MET alone, suggesting that treatment of IR with MET protects against a loss of mature neurons, though the underlying mechanisms were not explored. Overall, given that the underlying impairments of hyperactive mTORC1, inhibitory IRS‐1 phosphorylation, and neuronal protein content were recovered, our results provide evidence that MET treatment of IR is ineffective in improving the metabolic health in neurons.

As this study outlines, insulin resistance plays a direct role in regulating neuronal protein markers and morphology, and metformin neither blunts nor recovers these effects. However, the treatment and model used in this study are not fully comparable to T2D and AD; IR is not the only dysregulatory component present with these diseases, there is also a driving role for both mitochondrial impairment and inflammation that remains to be addressed. Whether IR alone impacts mitochondrial function and whether greater activation of inflammatory signaling, such as JNK, IKK*β*, and NF‐kB, plays a role in the impairments observed in this study, remains to be explored. Similarly, while some previous evidence does suggest a role for increased neurite size and length under conditions of stress in maintaining effective synaptic transmission despite a loss of synaptic number (DeKosky & Scheff, 1990), no measure of synaptic transmission was conducted to confirm this fact. As some evidence has shown that IR significantly impairs long‐term potentiation in rat hippocampal neurons (Grillo et al., 2015), it is imperative to determine whether our results, such as inhibitory IRS‐1 phosphorylation, would directly impact synaptic transmission. A closer investigation of the signaling involved in both the HI‐ and MET‐driven cell morphological enlargements should also be conducted to determine whether their impact on neurite outgrowth is compensatory for a reduced number of synaptic contacts, as was suggested by DeKosky and Scheff (1990) and Scheff et al. (1990), or whether it describes a separate underlying pathology associated with IR and MET treatments.

### 4.4 Study limitations and considerations

There were multiple considerations for our use of RA‐differentiated SH‐SY5Y rather than primary neuronal culture. Firstly, SH‐SY5Y cells are a human‐derived cell line that has been used widely in experimental neuroscience studies, including analysis of neuronal differentiation, metabolism, and function related to neurodegenerative processes, neurotoxicity, and neuroprotection (Biedler et al., 1973; Christensen et al., 2011; Encinas et al., 2002; Gimenez‐Cassina et al., 2006). Secondly, the SH‐SY5Y cell line possesses only neurons, rather than multiple cell types often seen within primary neuronal cultures. This allowed for the assessment of changes that specifically impact neurons, which lent itself toward this study's focus on investigating the impact of metformin and insulin signaling modulation on markers of synaptic function as a proof of concept. That is, does pharmacological modulation of insulin signaling in neurons modify synapses alone and can the addition of metformin improve neuron and synapse health. However, the presence of only neurons in the SH‐SY5Y model does not enable us to study the impact of other major cell types in the brain, such as microglia and astrocytes, and is thus a limitation of this study. Moving forward, multi‐cell models and co‐cultures, such as primary neuronal cultures or brain slice explants, will provide a better understanding of the physiological role that insulin resistance and metformin have on synaptic function in the presence of other cell types, including glia.

### 4.5 Conclusions

This study provides novel evidence that IR and MET impair synaptic and neuronal protein expression directly associated with neuronal health and that these impairments occur through distinct signaling pathways. We also show that, despite improving neurite and cell morphology, MET does not improve IR‐driven impairments in insulin signaling or synaptic protein expression. The neuronal markers explored in this study are known to be impaired with AD and their reduction following IR and MET treatments highlights the potential role that metabolic dysregulation might play in exacerbating AD progression. The results of this study highlight important risks associated with the use of metformin as an AD therapeutic and indicate that MET might not be effective in treating the metabolic impairments associated with IR in the brain. Further investigation should be conducted to effectively promote MET as a treatment to improve brain health and brain metabolism, particularly toward elucidating the exact mechanisms of metformin‐driven neuronal impairments as well as the impact of these impairments on synaptic transmission.

## AUTHOR CONTRIBUTIONS

Alex J. T. Yang conceived and designed the work, acquired data, interpreted the results, and drafted the manuscript. Ahmad Mohammad acquired data and revised the manuscript. Michael S. Finch acquired data and revised the manuscript. Evangelia Tsiani designed the work, interpreted the results, and revised the manuscript. Aleksandar Necakov designed the work, interpreted the results, and revised the manuscript. Gaynor Spencer designed the work, interpreted the results, and revised the manuscript. Rebecca E. K. MacPherson conceived and designed the work, interpreted the results, revised the manuscript, and takes accountability for all aspects of the work.

## FUNDING INFORMATION

This research was supported by the Alzheimer's Society of Brant, Haldimand Norfolk, Hamilton Halton. Alex Yang is funded by an NSERC Alexander Graham Bell Canada Graduate Scholarship‐Doctoral (CGS‐D).

## CONFLICT OF INTEREST STATEMENT

The authors declare no conflict of interest. The funders had no role in the design of the study; in the collection, analyses, or interpretation of data; in the writing of the manuscript, or in the decision to publish the results.

## INSTITUTIONAL REVIEW BOARD STATEMENT

This study was conducted in accordance with Brock University's ethical standards and Tri‐Council Policy Statement and has been approved by the Health Science Research Ethics Board of Brock University (17–397; 5/25/2022).

## Data Availability

The datasets used and/or analyzed during the current study are available from the corresponding author upon reasonable request.
